# Resource control of epidemic spreading through a multilayer network

**DOI:** 10.1038/s41598-018-20105-w

**Published:** 2018-01-26

**Authors:** Jian Jiang, Tianshou Zhou

**Affiliations:** 1 Research Center of Nonlinear Science, College of Mathematics and Computer Science, Wuhan Textile University, Wuhan, 430200 P.R. China; 2 Key Laboratory of Computational Mathematics, Guangdong Province, and School of Mathematics, Sun Yat-sen University, Guangzhou, 510006 P.R. China

## Abstract

While the amount of resource is an important factor in control of contagions, outbreaks may occur when they reach a finite fraction of the population. An unexplored issue is how much the resource amount is invested to control this outbreak. Here we analyze a mechanic model of epidemic spreading, which considers both resource factor and network layer. We find that there is a resource threshold, such that a significant fraction of the total population may be infected (i.e., an outbreak will occur) if the amount of resource is below this threshold, but the outbreak may be effectively eradicated if it is beyond the threshold. The threshold is dependent upon both the connection strength between the layers and their internal structure. We also find that the layer-layer connection strength can lead to the phase transition from the first-order phase to the continuous one or vice versa, whereas the internal connection can result in a different kind of phase transition (i.e., the so-called hybrid phase transition) apart from first-order and continuous one. Our results could have important implications for government decisions on public health resources devoted to epidemic disease control.

## Introduction

Epidemic spreading is typically a dynamical process. In previous studies^1–9^, the spreading was considered to be in single or monoplex networks, which is apparently a simplification since in reality, epidemic diseases spread often through multiple channels, e.g., through different ways of human travelling (airports, train, bus, etc.), just as information may be diffused through different online social mediums such as Twitter and Facebook. Moreover, many epidemic spreading processes may simultaneously occur through multiple routes, e.g., sexually transmitted diseases may spread both in homosexual and in heterosexual networks. Compared to single networks, multilayer networks can explicitly consider the diversity of multi-channel connection and may well describe a system interconnected through different categories of connection, where each channel is represented by a layer network, which allows various possible kinds of connections between nodes and interactions between layer networks. Thus, advantages of the intrinsic characteristics of multilayer networks are in providing a natural way to extend and improve the understanding of spreading dynamics in real-world complex networked systems^10–13^.

Recently, Saumell-Mendiola *et al*. analyzed the effect of a multilayer coupled network on disease spreading using a classic susceptible-infected-susceptible (SIS) model^14^. They found that few inter-layer edges may cause a global endemic state in the entire network rather than in a single-layer network, and that the strength of degree-degree correlation between layers can decrease the epidemic threshold. By studying the susceptible-infected-recovery (SIR) epidemic spreading through a multilayer network, Dickison *et al*. revealed that in the case of strong coupling, an epidemic disease can spread from one layer to another at a critical infection strength below which the spread does not occur, while in the case of weak coupling, the epidemic spreads only within one layer when the critical infection strength is not reached^15^. Wei *et al*. proposed a model for cooperative spreading processes on interacting two-layer networks, and they proved that the epidemic threshold of the interaction is decreasing^16^. Velasquez-Rojas *et al*. investigated how opinion formation and disease spreading processes are mutually affected in multiplex networks^17^. Jovanovski *et al*. developed a SIS model of multiple contagions, which incorporates different spreading channels and disease mutations in a multilayer network^18^. Some authors considered other aspects of epidemic spreading in multilayer networks, including disease localization^19^, the effect of opinion exchanges on vaccination^20^, the interaction between the spreading of epidemics and awareness information^21^, and so on. In a short, the existing studies paid more attention on the impact of multilayer network structure or on the interaction between different epidemic dynamics.

In a realistic scenario, however, the available social and medical resources to treat diseases or to prevent their spreading are usually limited, and have been verified to be an important factor impacting disease spreading. This factor is directly relevant to the problem of designing optimal strategies for suppressing the epidemic outbreak to the greatest extent and for minimizing the prevalence once the epidemic outbreak has happened. Very recently, Shai *et al*. studied a model of a constrained SIR process on coupled networks where nodes are limited to interact with a maximum number of neighbors^22^. They found that in the absence of resource constraint, positive correlation coupling leads to a lower epidemic threshold than a negative one. In the presence of constraint, however, the spreading is less efficient in the former case than in the latter case. Similar results were found in ref.^23^, where Chen *et al*. assumed that the recovery rate is positively correlated with the average resource devoted to each infected individual and observed that the disease outbreak can be effectively eradicated, only when the amount of resource is above a critical value^24^. In addition, Chen^25^ and Enyioha^26^ presented a distributed resource allocation strategy to control an epidemic outbreak, respectively. Bottcher *et al*. considered that the recovery of sick individuals depends on the availability of healing resources that are generated by a healthy population, finding that epidemics spiral out of control into explosive spread if the cost of recovery is beyond a certain critical value^27^. In spite of these endeavors, how resource affects epidemic spreading in a multilayer network remains unexplored.

**Figure 1:**
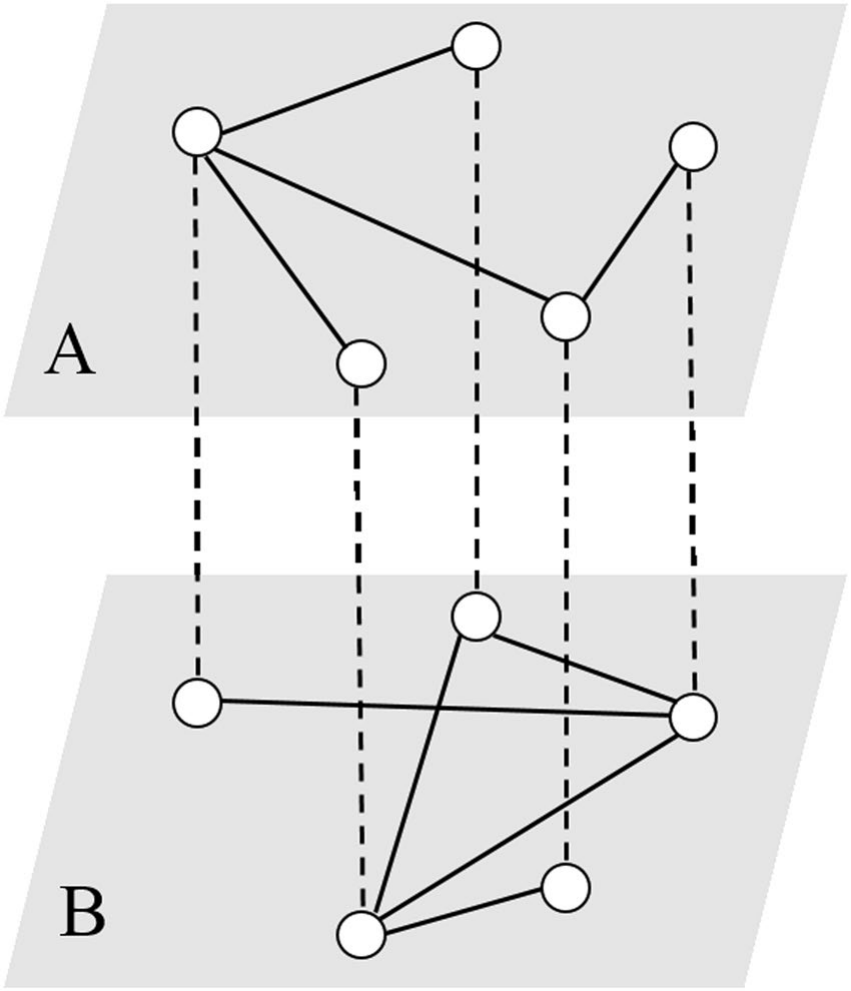
Schematic illustration of a two-layer network. The topologies in layers *A* and *B* may be different, but each node in one layer is connected to its counterpart in the other layer. Dashed and solid lines represent interlayer and intralayer connections, respectively.

In the present paper, inspired mainly by the work of ref.^24^, we study the effect of resource amount on epidemic control using a modified SIS model. Specifically, this model considers a two-layer network consisting of two random networks *A* and *B* sharing the same set of nodes, as shown in Fig. 1, where the infected nodes in one subnetwork can pass the disease to their neighbors in another subnetwork until they finally recover. We assume that the recovery rate for infected individual is not a constant but a variable over time depending on the average resource that each infected node gets. By model analysis, we show that a critical resource amount is needed to suppress the disease spreading. In addition, we find interesting dynamical phenomena, e.g., given a resource amount, the spreading process goes through a first-order phase transition if the infection strength between layers is weak, but a continuous phase transition if the infection strength becomes strong, and the topological structure within a layer network can lead to a multi-phase behavior apart from first-order and continuous phase transitions.

## Methods

As pointed out in the introduction, resource invested in curing an infected population plays a critical role in containing the epidemic spreading. In order to reveal the essential mechanism of how the resource affects control of epidemic diseases spreading through multiplex networks, we consider a two-layer network where each subnetwork is modelled by a discrete set of SIS equations. In both layer networks, each node may switch between the susceptible (S) state and the infected (I) state, and the links between the nodes represent the connections along which the disease can propagate. At each time step, the nodes with state S can be infected simultaneously by intra-layer and inter-layer infected nodes, but the infected nodes may recover to susceptible nodes.

Let *β*_1_ (*β*_2_) represent the transmission probability in network *A* (*B*) that an infected node spreads the disease to a neighbor to which it shares a link, *γ*_1_ (*γ*_2_) the probability of transmission from a node in *B* (*A*) to a node in *A* (*B*), i.e., each of *γ*_1_ and *γ*_2_ represents a connection strength between the two layers, and *μ*_1_(*t*) (*μ*_2_(*t*)) the recovery probability in network *A* (*B*) that an infected node recovers to a susceptible node at time step *t*. If *γ*_1_ = *γ*_2_ = 0, the disease spreads only in a single subnetwork. In this case, the model of epidemic spreading in the multiplex network is reduced to that in a single-layer network. If the only one of *γ*_1_ and *γ*_2_ is non-zero, then the disease spreading is unidirectional in the two-layer network. If both are non-zero, the spreading is bidirectional between the two layers. Denote by *p*_1,*i*_(*t*) and *p*_2,*i*_(*t*) the probabilities that a node *i* in networks *A* and *B* is at the infected state at time *t*, respectively. Then, the time evolutions of *p*_1,*i*_(*t*) and *p*_2,*i*_(*t*) can be described by the following discrete dynamical equations^28,29^:

1a $${p}_{1,i}(t+1)=[1-{p}_{1,i}(t)]\,[1-{q}_{1,i}(t)]+[1-{\mu }_{1}(t)]{p}_{1,i}(t)+{\gamma }_{1}\,{p}_{2,i}(t)[1-{p}_{1,i}(t)],$$

1b $${p}_{2,i}(t+1)=[1-{p}_{2,i}(t)]\,[1-{q}_{2,i}(t)]+[1-{\mu }_{2}(t)]{p}_{2,i}(t)+{\gamma }_{2}\,{p}_{1,i}(t)[1-{p}_{2,i}(t)],$$

where *q*_1,*i*_(*t*) and *q*_2,*i*_(*t*) are the probabilities that node *i* is not infected by any of its neighbors in *A* and *B* respectively, that is,

2a $${q}_{1,i}(t)=\prod _{j=1}^{N}[1-{\beta }_{1}{a}_{ij}{p}_{1,j}(t)],$$

2b $${q}_{2,i}(t)=\prod _{j=1}^{N}[1-{\beta }_{2}{b}_{ij}{p}_{2,j}(t)].$$

In Eq. (2), (*a*_*ij*_) and (*b*_*ij*_) represent the adjacency matrices of networks *A* and *B* respectively, and each of *a*_*ij*_ and *b*_*ij*_ takes value 1 when nodes *i* and *j* share a common link and value 0 otherwise. *N* is the size of network *A* or *B*.

In the first line of Eq. (1a), the first term on the right-hand side, 1 − *p*_1,*i*_(*t*), is the probability that node *i* is susceptible whereas 1 − *q*_1,*i*_(*t*) is the probability that node *i* is infected by at least a neighbor in layer *A*, the second term represents the probability that node *i* is infected at time *t* and does not recover, and the third term takes into account the probability that the susceptible node *i* is infected by the counterpart node in layer *B*. Similar interpretations hold for Eq. (1b). The final infected population sizes in *A* and *B* are defined as fractions of the total population respectively, that is,

3 $${\rho }_{i}(t)=\frac{1}{N}\sum _{j=1}^{N}{p}_{i,j}(t),i=1,2.$$

It is particularly emphasized that two recovery probabilities *μ*_1_(*t*) and *μ*_2_(*t*) in Eq. (1), which were originally assumed as constants in the traditional SIS model, are currently assumed as two time-varying parameters and depend on the average resource *R* that each infected node would get. Since the resource that a country uses to epidemic control is limited, *R* should be related to the financial power of this country in practical control of epidemic diseases. Furthermore, we assume that *R* is a percentage of the fully economic output of a country, evaluated between 0 and 1. According to the experimental results of ref.^24^, we may assume that two recovery probability functions take the forms

4 $${\mu }_{i}(t)={e}^{-c{\rho }_{i}(t)/R},i=1,2,$$

where *c* is a control coefficient to decide the relative importance of *ρ*_*i*_(*t*) and *R*, and we will set *c* = 1 for simplicity. Since each recovery probability for infected individuals, *μ*_*i*_(*t*), decreases with the decrease of *R*, and is close to 0 when *R* → 0, this assumption is reasonable (remark: here we consider only the case that epidemic diseases are so severe or fatal that the infected people cannot be recovered without drug treatment, implying that the case that people can self-heal without medicine is not considered). Note that when *R* → 0, the present epidemic model is reduced to the traditional SI model.

In order to reveal the essential mechanism of how the average resource amount impacts epidemic control, we consider a special case in the following, that is, the two-layer network is assumed to consist of two random regular networks with uniform degree *k*_1_ and *k*_2_, respectively. In this case, Eq. (2) becomes

5a $${q}_{1,i}(t)={[1-{\beta }_{1}{p}_{1,j}(t)]}^{{k}_{1}},$$

5a $${q}_{2,i}(t)={[1-{\beta }_{2}{p}_{2,j}(t)]}^{{k}_{2}},$$

Since the nodes in a random regular network are indistinguishable from each other, it follows from Eq. (3) that *p*_*i*,*j*_(*t*) = *ρ*_*i*_(*t*) (*i* = 1, 2, *j* ∈ 1, & …; *N*), which is equivalent to the mean field approximation. When the epidemic spreading process reaches steady state, and if we denote by *ρ*_1_ and *ρ*_2_ the final infected population sizes for *A* and *B* at steady state respectively, Eq. (1) will be reduced to

6a $$(1-{\rho }_{1})[1-{(1-{\beta }_{1}{\rho }_{1})}^{{k}_{1}}+{\gamma }_{1}{\rho }_{2}]-{\rho }_{1}{e}^{-{\rho }_{1}/R}=0,$$

6b $$(1-{\rho }_{2})[1-{(1-{\beta }_{2}{\rho }_{2})}^{{k}_{2}}+{\gamma }_{2}{\rho }_{1}]-{\rho }_{2}{e}^{-{\rho }_{2}/R}=0,$$

From Eq. (6), we can establish the following relationship between the final infected population sizes in two subnetworks

7 $${\rho }_{2}=\frac{{\rho }_{1}{e}^{-{\rho }_{1}/R}}{{\gamma }_{1}(1-{\rho }_{1})}-\frac{1-{(1-{\beta }_{1}{\rho }_{1})}^{{k}_{1}}}{{\gamma }_{1}}\equiv A({\rho }_{1}).$$

Furthermore, we can derive the following algebraic equation of ${\rho }_{1}$ from Eq. (6) combined with Eq. (7)

8 $${e}^{-A({\rho }_{1})/R}-[1+{\gamma }_{2}{\rho }_{1}-{(1-{\beta }_{2}A({\rho }_{1}))}^{{k}_{2}}](\frac{1}{A({\rho }_{1})}-1)=0$$

In the small limit of resource amount *R*, Eq. (8) is further reduced to the following algebraic equation of *ρ*_1_

9 $${(1+{\gamma }_{2}{\rho }_{1})}^{1/{k}_{2}}=1+\frac{{\beta }_{2}}{{\gamma }_{1}}[1-{(1-{\beta }_{1}{\rho }_{1})}^{{k}_{1}}]$$

which has a nontrivial solution under some conditions of system parameters, apart from the trivial solution *ρ*_1_ = 0. Note that a nontrivial *ρ*_1_ implies a nontrivial *ρ*_2_ according to Eq. (7).

In our numerical analysis, except that the effect of *R* is considered, the effect of the only one connection strength (i.e., *γ*_1_) is considered due to symmetry of our model. Therefore, it is unnecessary to additively consider the case of *γ*_2_.

## Results

### A critical resource amount for epidemic control

**Figure 2:**
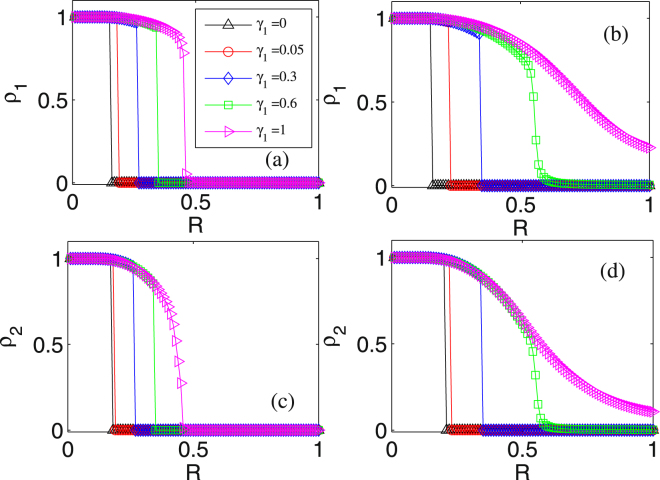
Influence of resource amount on the fraction of the infected population: (**a**,**b**) dependence of *ρ*_1_ on *R* for different values of *γ*_1_, where (**a**) *γ*_2_ = 0.08 and (**b**) *γ*_2_ = 0.2 are fixed; (**c**,**d**) dependence of *ρ*_2_ on *R* for different values of *γ*_1_, where (**c**) *γ*_2_ = 0.08 and (**b**) *γ*_2_ = 0.2 are fixed. The other parameter values are set as *ρ*_1_(0) = *ρ*_2_(0) = 0.1, *k*_1_ = *k*_2_ = 30, and *β*_1_ = *β*_2_ = 0.02.

Here we investigate the influence of resource amount (*R*) on two final infected subpopulations quantified by *ρ*_1_ and *ρ*_2_. Numerical results are shown in Fig. 2, where the values of parameters *k*_1_, *k*_2_ and *β*_1_, *β*_2_ are fixed. Specifically, Fig. 2(a,b) shows the dependence of *ρ*_1_ on *R* for several different values of *γ*_1_ in two cases of *γ*_2_: a small connection strength, e.g., *γ*_2_ = 0.08 to which Fig. 2(a) corresponds, and a large connection strength, e.g., *γ*_2_ = 0.2 to which Fig. 2(b) corresponds. From these two panels, we observe that in both cases, *ρ*_1_ is a monotonically decreasing function of *R* but the change tendency is different. In the case of small *γ*_2_, the curves for the dependence of *ρ*_1_ on *R* always exhibit discontinuity whatever *γ*_1_. In contrast, in the case of large *γ*_2_, the curves for the dependence of *ρ*_1_ on *R* exhibit first discontinuity for small values of *γ*_1_ and then continuity for large values of *γ*_1_.

It is interesting that in the case of small *γ*_2_, e.g., *γ*_2_ = 0.08 for Fig. 2(a), there is a critical value of *R*, denoted by *R*_*c*_, such that at this critical threshold, *ρ*_1_ has a sudden transition from non-zero value to zero, which is a signature of the first-order phase transition. Specifically, if *R* < *R*_*c*_, then the disease will spread to a large fraction of the population in network *A*, whereas if *R* < *R*_*c*_, then it will be well contained within a negligible fraction of the population. Moreover, the critical threshold *R*_*c*_ is dependent on the connection strength between layers, *γ*_1_. Precisely, the larger *γ*_1_ the larger *R*_*c*_. However, a larger *γ*_1_ means that the disease is more easily transmitted from layer *B* to layer *A*, indicating that it is needed to invest the more resource to contain the disease spreading in layer *A*. From panel (b) which corresponds to a large *γ*_2_, we observe that *ρ*_1_ undergoes a significant change from the first-order phase transition to continuous phase transition, i.e., *ρ*_1_ decreases continuously from non-zero to a finite value with the increase of *R*. A possible reason for this change is that the more average resource amount is needed to suppress epidemic spreading since increasing *γ*_1_ or *γ*_2_ means that the disease spreads more easily from layers *A* to *B* or vice versa. When *γ*_1_ is small, the disease is effectively controlled since the system behaves as the first-order phase transition with a large *R*_*c*_, but when *γ*_1_ is large, a small fraction of population is still infected as no more resource amount is supplied.

In panels (c) and (d), *ρ*_2_ has the change tendency similar to *ρ*_1_. However, the values of *ρ*_2_ and *ρ*_1_ are dependent on *γ*_2_ and *γ*_1_ when the other parameters are kept the same. If *γ*_2_ > *γ*_1_, which means that the disease is more easily transmitted from layers *A* to *B* than from layers *B* to *A*, then *ρ*_2_ > *ρ*_1_ with the same *R*. Otherwise, *ρ*_2_ < *ρ*_1_ with the same *R*. For example, for the case of *γ*_1_ = 1.0 shown by the pink symbols in the figure, it is apparent that the value of *ρ*_2_ in panel (c) is smaller than that of *ρ*_1_ in panel (a) with the same resource amount.

In a word, from the viewpoint of dynamics, parameter *R* can induce first-order and continuous phase transitions of *ρ*_1_ and *ρ*_2_, depending on *γ*_1_ and *γ*_2_, implying that investing a suitable amount of resource can well control epidemic spreading, which is a main result of this paper.

### Influences of layer-layer connection strengths on infected populations at fixed amounts of resource

**Figure 3:**
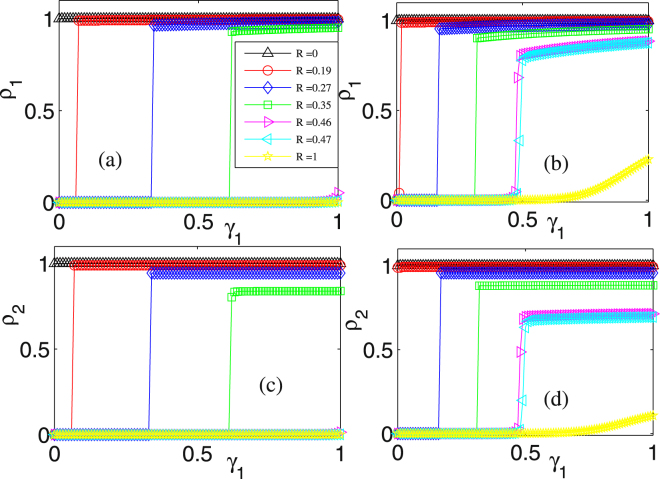
Influence of layer-layer connection strength on the fraction of the infected population for different resource amounts: (**a**,**b**) *ρ*_1_ vs *γ*_1_, where (**a**) *γ*_2_ = 0.08 and (**b**) *γ*_2_ = 0.2 are fixed; (**c**,**d**) *ρ*_2_ vs *γ*_1_, where (**c**) *γ*_2_ = 0.08 and (**d**) *γ*_2_ = 0.2 are fixed. The other parameter values are set as *ρ*_1_(0) = *ρ*_2_(0) = 0.1, *k*_1_ = *k*_2_ = 30, and *β*_1_ = *β*_2_ = 0.02.

The influence of layer-layer connection strength on the fraction of the infected population for different resource amounts is shown in Fig. 3. Panel (a) corresponds to *γ*_2_ = 0.08. If *R* = 0, then the recovery rate for each individual is zero, implying that the final infected population *ρ*_1_ is equal to one. If *R* > 0, *ρ*_1_ increases discontinuously with the increase of *γ*_1_. For a given *R* and at the critical threshold *γ*_1*c*_,*ρ*_1_ jumps abruptly from zero to a non-zero value, behaving as the first-order phase transition. If the resource amount is large enough, e.g., up to *R*_*c*_ = 0.47 represented by the cyan symbols in panel (a), *ρ*_1_ is close to zero, implying that no disease spreads regardless of *γ*_1_. In panel (b) where *γ*_2_ = 0.2 that means that the connection strength between layers has been enhanced in contrast to panel (a), *ρ*_1_ increases from zero to a finite value at smaller *γ*_1*c*_ compared to the case of panel (a). In addition, even though *R* > *R*_*c*_ = 0.47, *ρ*_1_ is no longer zero but goes through a continuous phase transition and grows to a finite value as *γ*_2_ continuously increases. Panels (c) and (d) show similar influence of *γ*_1_ on *ρ*_1_ as in panels (a) and (b). When *γ*_1_ is larger than *γ*_2_ = 0.08 (panel (c)) or *γ*_2_ = 0.2 (panel (d)), the value of *ρ*_2_ is smaller than that of *ρ*_1_, or vice versa. The reason for this change is the same as in the cases of panels (c) and (d) in Fig. 2

In a word, for a given resource amount, parameter *γ*_1_ or *γ*_2_ can induce first-order phase transitions of *ρ*_1_ and *ρ*_2_, implying the importance of the connection strengths between layers in controlling the spreading of epidemic diseases, in accordance with the common sense.

### The joint influences of resource amount and layer-layer connection strengths on infected populations

**Figure 4:**
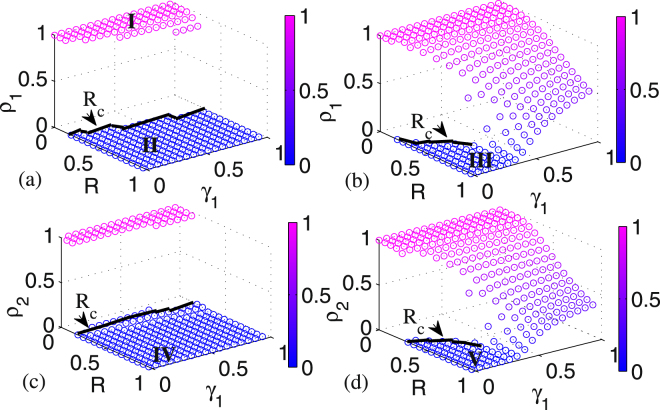
The joint influence of resource amount and layer-layer connection strength on the fraction of the infected population: (**a**,**b**) dependence of *ρ*_1_ on both *R* and *γ*_1_, where (**a**) *γ*_2_ = 0.01 and (**b**) *γ*_2_ = 0.4 are fixed; (**c**,**d**) dependence of *ρ*_2_ on both *R* and *γ*_1_, where (**c**) *γ*_2_ = 0.01 and (**d**) *γ*_2_ = 0.4 are fixed. In (**a**–**d**), the boundary for the critical threshold of *R*_*c*_ is indicated. The other parameter values are set as *ρ*_1_(0) = *ρ*_2_(0) = 0.1, *k*_1_ = *k*_2_ = 30, and *β*_1_ = *β*_2_ = 0.02.

Figure 4 shows the joint influence of resource amount *R* and layer-layer connection strength *γ*_1_ on the final infected population *ρ*_1_. In panel Fig. 4(a) where *γ*_2_ = 0.01 is fixed, it is obvious that when the resource amount is inadequate, e.g., it is less than the critical threshold *R*_*c*_ indicated by black line, *ρ*_1_ is in a region of the active state^30,31^ where the disease could spread out in a high fraction of population, as shown in region I marked by pink color in panel Fig. 4(a). Once *R* > *R*_*c*_, *ρ*_1_ will directly jump to a region of the absorbing state^30,31^ where the disease could be die out eventually, as shown in region II marked by blue color. The epidemic spreading process is characterized by the first-order phase transition, which does not change with *γ*_1_. In panel Fig. 4(b) where *γ*_2_ is set as 0.4, we find that when *γ*_1_ < 0.3, the spreading process exhibits the behavior of a first-order phase transition, but when *γ*_1_ > 0.3, the pattern of spreading is changed to a continuous phase transition. Moreover, for a given resource amount *R*, a larger *γ*_1_ corresponds to a higher final infected population. These results are obtained by increasing *γ*_2_ that means that the disease spreads more easily from layer *B* to layer *A*.

In panels Fig. 4(c) and (d), *ρ*_2_ has a similar change tendency like *ρ*_1_. With a small *γ*_2_ in panel (c), *ρ*_2_ behaves as the first-order phase transition. However, with a large *γ*_2_ in panel (d), the pattern of *ρ*_2_ changes from the first-order phase transition to the continuous phase transition when *γ*_1_ is larger than 0.3. Furthermore, in panel (c), as *γ*_2_ is less than the main available range of *γ*_1_, the region with absorbing state in layer *B* (region IV shown in panel (c)) is larger than that in layer *A* (region II shown in panel (a)). Similarly, in panel (d), if *γ*_1_ < *γ*_2_ = 0.4, then the region with absorbing state in layer *B* (region V shown in panel (d)) is smaller than that in layer *A* (region III shown in panel (b)). However, if *γ*_1_ > *γ*_2_ = 0.4, then the final infected population size in layer *B* is smaller than that of layer *A*.

**Figure 5:**
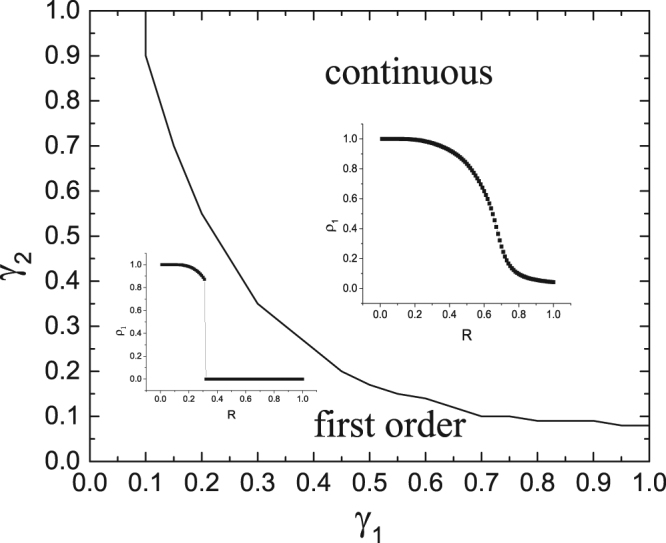
Phase regions of the system in the (*γ*_1_, *γ*_2_) plane, where a different sub-region corresponds to one different mode of the relationship between *ρ*_1_ and *R*. The other parameter values are set the same as Fig. 4.

The above findings show that both the resource amount and the layer-layer connection strength can dramatically impact the behavior of the epidemic spreading process, that is, constraining the resource amount may result in the first-order phase of epidemic spreading, and the layer-layer connection strength may change the pattern of phase transition. As shown in Fig. 5, for the same value of *γ*_2_, as *γ*_1_ increases, the spreading process in layer *A* evolves from the first-order phase transition to the continuous phase transition. In particular, when *γ*_1_ or *γ*_2_ is small, only the first-order phase transition takes place.

### The joint influences of resource amounts and subnetworks’ topological degrees on infected populations

**Figure 6:**
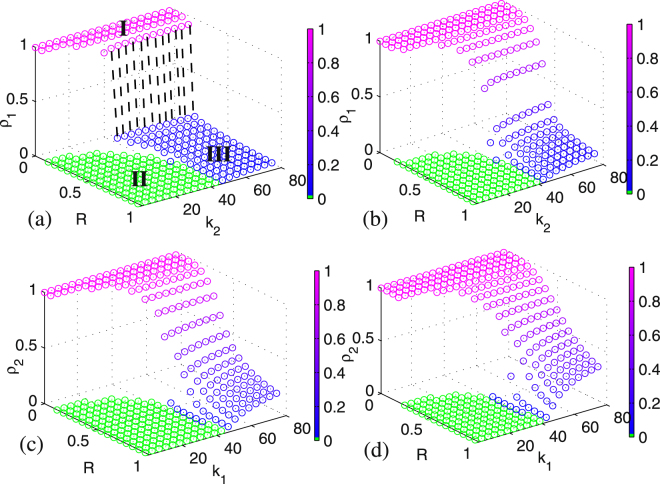
The joint influence of resource amount and topological degree on the fraction of the infected population: (**a**,**b**) dependence of *ρ*_1_ on both *R* and *k*_2_, where (**a**) *k*_1_ = 4 and (**b**) *k*_1_ = 30 are fixed; (**c**,**d**) dependence of *ρ*_2_ on both *R* and *k*_1_, where (**c**) *k*_2_ = 4 and (**d**) *k*_2_ = 30 are fixed. In (**a**), three different regions corresponding to three different kinds of phases are indicated. The other parameter values are set as *ρ*_1_(0) = *ρ*_2_(0) = 0.1, *β*_1_ = *β*_2_ = 0.02, and *γ*_1_ = 0.08, *γ*_2_ = 0.4.

This subsection analyzes the impact of the connection strength within a layer network on the disease spreading. Note that a larger topological degree means a stronger connection strength within the layer network, which facilitates epidemic spreading in the full system. Figure 6 shows the joint influence of resource amount *R* and topological degree on the fraction of the infected population. In panel (a) where *k*_1_ = 4 is fixed, *ρ*_1_ may exhibit a first-order phase transition if *k*_2_ is less than 30. That is, *ρ*_1_ jumps abruptly from region I to region II marked by green color, meaning that the disease can be well controlled in a negligible fraction of population. Along with the increase of *k*_2_, the spreading process evolves from a first-order phase transition to another (i.e., the so-called hybrid phase transition), where *ρ*_1_ jumps sharply from region I to region III marked by blue color, meaning that the disease can be effectively controlled in a very low fraction of population.

In panel Fig. 6(b) where *k*_1_ is set as 30, an enlarged connection strength changes the pattern of the spreading process in layer *A*, which evolves from a hybrid phase transition to a continuous phase transition in the range of *k*_2_ > 40. In the range of *k*_2_ < 40, however, the spreading process still undergoes the first-order phase transition. In panel (c) where *k*_2_ = 4 is fixed, behavior of *ρ*_2_ is similar to that of *ρ*_1_ in panel (b) rather than in panel (a). A possible reason for this change is that *γ*_2_ is set much larger than *γ*_1_ in our simulation, resulting in a continuous phase transition of *ρ*_2_, not a hybrid phase transition at a large *k*_1_. When *k*_2_ increases to 30 in panel (d), *ρ*_2_ has similar spreading dynamics as in panel (c), but an enlarged connection strength can make both the values of critical threshold *R*_*c*_ at a small *k*_1_ and the final infected population at a large *k*_1_ become larger. The multi-phase behavior of the spreading process hints that there is a strong influence in spreading dynamics between two layer networks.

**Figure 7:**
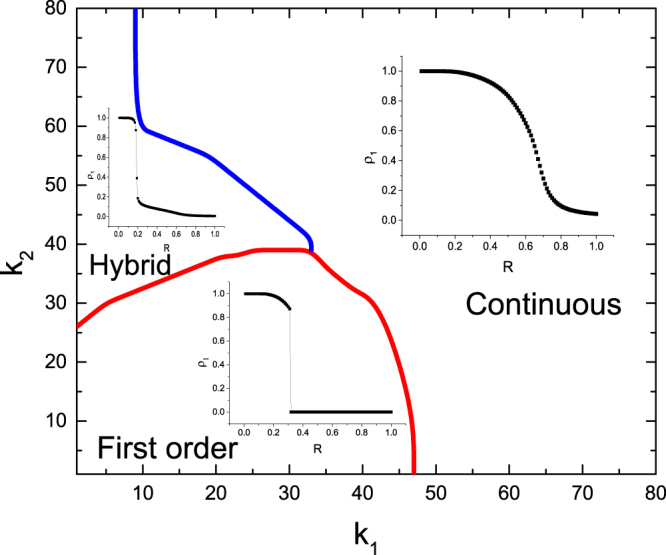
Phase regions in the (*k*_1_, *k*_2_) plane, where a different sub-region corresponds to a different mode of the relationship between *ρ*_1_ and *R*. The other parameter values are set as in Fig. 6.

In order to more clearly show the multi-phase behavior of epidemic spreading in Fig. 6, we plot the phase regimes of the system for different internal connection strength *k*_1_ and *k*_2_ in Fig. 7. We observe that there is an abrupt switch between the hybrid phase transition and the first-order phase transition at the critical threshold *R*_*c*_. The final infected population is a positive value in the former, but zero in the latter. Notice that at a small *k*_1_, three kinds of phase transitions could coexist, but for a large *k*_1_, only the continuous phase transition exists.

## Conclusions and simple Discussions

In contrast to previous studies that considered only single layer networks and did not consider resource factor, the present work considered not only resource amount invested to epidemic control but also a two-layer network consisting of two random networks sharing the same set of nodes. By analyzing a toy model (see Eq. (1)), we have found interesting dynamical phenomena, e.g., resource amount parameter *R* can induce not only first-order phase transitions but also continuous phase transitions of the fractions of the infected populations *ρ*_1_ and *ρ*_2_, depending on the connection strengths *γ*_1_ and *γ*_2_; parameter *γ*_1_ or *γ*_2_ can only induce first-order phase transitions of *ρ*_1_ and *ρ*_2_ for a given *R*; the cooperation between *R* and *γ*_1_ or *γ*_2_ can induce both first-order and continuous phase transitions of *ρ*_1_ and *ρ*_2_; and the cooperation between *R* and *k*_1_ or *k*_2_ can induce three different kinds of phase transitions of *ρ*_1_ and *ρ*_2_: first-order, continuous and hybrid. In particular, we have found that there is a critical value of resource amount (*R*_*c*_) such that when *R* < *R*_*c*_, the disease can spread to a large fraction of the population in subnetwork *A*, whereas when *R* > *R*_*c*_, the epidemic spreading can be well contained in a negligible fraction of the population. These results not only indicate that resource investment is a pivotal factor of controlling epidemic spreading but also could have important implications for government decisions on public health resources devoted to epidemic control.

Although all the results obtained in this paper are qualitative, independent of the choice of model parameters, our model used for analysis made simplification in many aspects. For example, in a more realistic case, diseases would spread through more than two layer networks whereas our model considered only a two-layer network; a different subnetwork would have the different number of nodes but our model assumed that the number of nodes in both subnetworks is the same; and each node in a different subnetwork would have a different topological degree while our model assumed that it is the same. In spite of these, we are expecting that the results obtained here are kept qualitatively unchanged.

In addition, our model neglected the effect of noise. As is well known, the noise exists extensively in systems of epidemic disease spreading (in fact, the noise is inevitable during the process of epidemic spreading, due to the uncertainty of either spreading factors or environments). It is also well known that the noise can induce stochastic switching between different states of a dynamical system^32–34^. A naturally arising question is how the noise affects epidemic spreading in the case that the factor of resource investment is considered. It seems to us that none investigates such a question although it is worth study.

Finally, we point out that while resource and network layer are two unneglectable factors, the framework of modeling and analysis here would provide a paradigm for studying other similar real complex networks.
